# Effect of addition of adjuvants on physical and chemical characteristics of Bt bioinsecticide mixture

**DOI:** 10.1038/s41598-019-48939-y

**Published:** 2019-08-29

**Authors:** Cicero Antônio Mariano dos Santos, Renata Thaysa da Silva Santos, Jaqueline Franciosi Della’Vechia, Fabiano Griesang, Ricardo Antônio Polanczyk, Marcelo da Costa Ferreira

**Affiliations:** 1 Department of Plant Health, São Paulo State University (Unesp), School of Agricultural and Veterinarian Sciences, Jaboticabal, 14884-900 Brazil; 2 Department of Plant Production, São Paulo State University (Unesp), School of Agricultural and Veterinarian Sciences, Jaboticabal, 14884-900 Brazil

**Keywords:** Bacterial techniques and applications, Environmental sciences

## Abstract

*Bacillus thuringiensis* (Bt) is the main bacterium used in the formulation of bioinsecticides because it produces toxins and spores that are toxic to several orders of insects. The efficacy of Bt bioinsecticide is influenced by the quality of its application. The association with other crop protection products, such as adjuvants, can affect the physical and chemical parameters of the mixture. This study evaluated the physical and chemical parameters, volume median diameter (VMD), uniformity coefficient of droplets (SPAN), percentage of volume in drift droplets (%V <100 µm), contact angle, surface tension, potential of hydrogen (pH) and electrical conductivity (E.C.) of Bt bioinsecticides in concentrated suspension (SC), and wettable powder (WP) formulations associated with adjuvants. The largest droplet diameter and smallest values of drift droplets were found in the WP formulation with lower drift potential. The addition of mineral oil and surfactant to the mixtures of bioinsecticide reduced contact angle values and surface tension of the droplets, resulting in greater spreading of droplets in leaves. The addition of lecithin and propionic-acid-based adjuvants lowered the pH in both formulations. The adjuvants used in this study affected the physical and chemical characteristics of the mixtures, improving or impairing the quality of Bt bioinsecticide applications.

## Introduction

*Bacillus thuringiensis* (Bt) produces toxins, which are harmful to several orders of insects, mites, nematodes, and protozoa^1^, during the vegetative and sporulation phases^2–4^. Bt bioinsecticides have two important virulence factors: Cry toxins and spores^2–4^. Their action modes include the following stages: (1) ingestion of the spore-crystal complex by the susceptible insect, (2) solubilization and processing of the toxin, (3) binding to the receptor, (4) membrane insertion, (5) pore formation, and (6) cell lysis^5^. The latter causes septicemia at the end of the infection process when the pH of the insect’s intestine is reduced, making germination possible^6^.

The efficiency of bioinsecticides is strongly influenced by the quality of application, since the greater the leaf coverage of the spray, the more likely for target insects to ingest lethal doses of the biological agent^7^. Bt bioinsecticides are diluted with water prior to spray application. The spraying may be isolated or associated with other plant protection products such as adjuvants^7,8^. The addition of adjuvants to crop protection mixtures is becoming more important because of improvements to the crop protection product application process, such as droplet and chemical molecule protection, drift reduction, evaporation, and improved droplet spreading on leaf surfaces of plants^9–11^.

Moreover, adjuvants can change important physical and chemical characteristics of crop protection solutions, such as hydrogenation potential (pH), electrical conductivity (E.C.), and surface tension. Adjuvants can also affect both the contact angle and formation of droplets, influencing the volume median diameter (VMD), uniformity coefficient (SPAN) and percentage of droplets subject to drift (%V < 100 µm). The pH of Bt bioinsecticide mixtures is important because values below or above the ideal for Bt mixtures affect the number of viable spores. Low pH bioinsecticide spraying causes the inactivation of the toxin by heating, while the high pH provides protein solubilization affecting the control potential of insect pests^12,13^. Reduced surface tension and droplet contact angle results in increased droplet spreading and greater coverage of the contact surface with the target, increasing the probability of contact with the target insects and consequently, control efficiency^14–16^.

Few studies have addressed interactions of Bt bioinsecticides with adjuvants, especially the effect of this interaction on the efficiency and persistence of Bt under adverse environmental conditions^17–19^. Adjuvants can inhibit or stimulate growth, reproduction, and mutations, or even inactivate microorganisms, reducing virulence for a particular pest, because of physical and chemical interactions in a mixture^20^. The aim of this study, therefore, is to evaluate the effect of adjuvant addition on the physical and chemical characteristics of Bt bioinsecticide mixtures.

## Results

### Spectrum of spray droplet size

Three factors affecting target coverage of the sprayed mixture were considered for evaluation of the droplet size spectrum: the volume median diameter (VMD), uniformity coefficient of droplets (SPAN) and percentage of drift droplets (%V < 100 µm).

**Figure 1:**
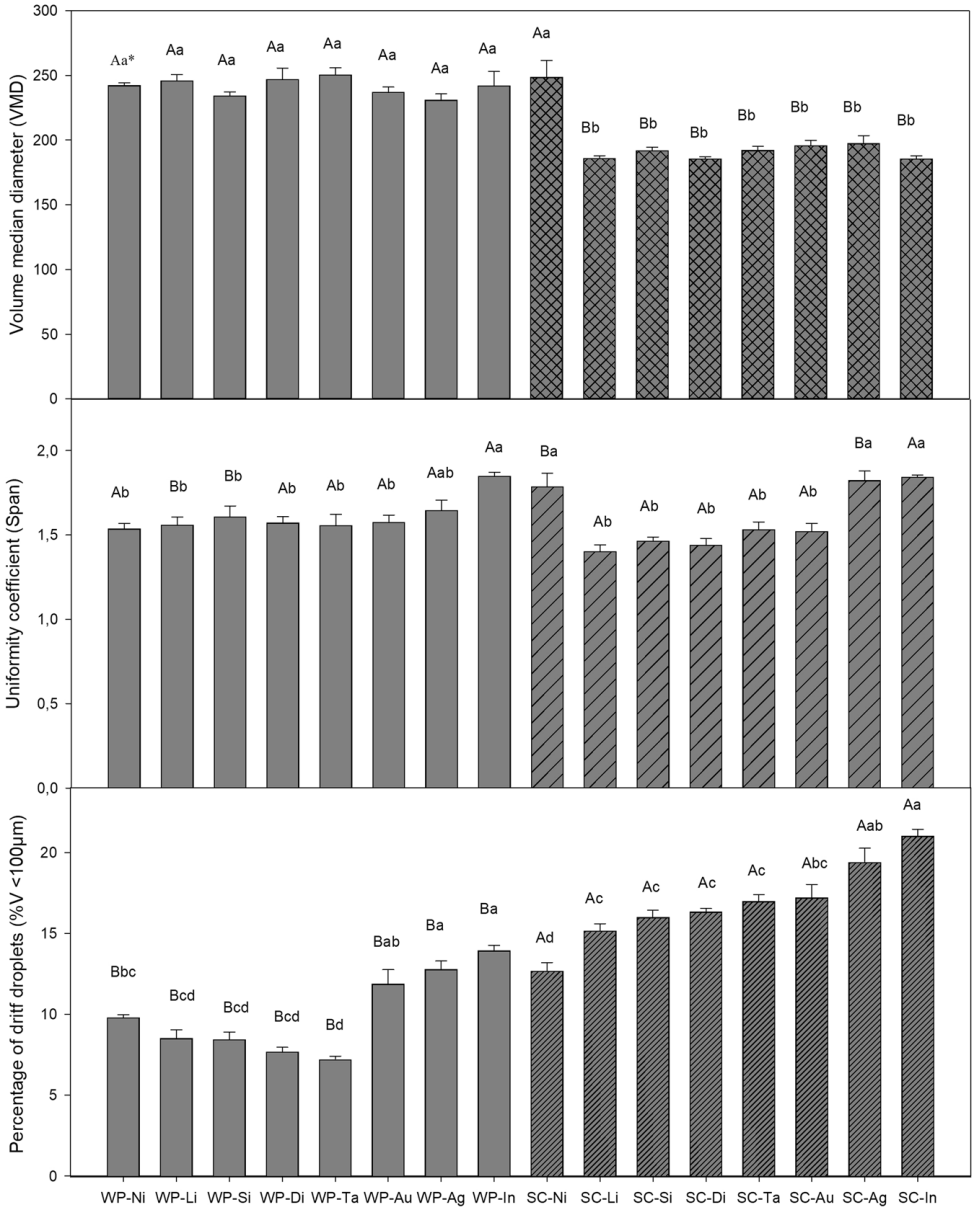
Volume median diameter (VMD) values. Coefficient of uniformity (SPAN) and percentage of droplets less than 100 µm (<100) in different mixtures. Legend: WP-Ni: WP + Mineral Oil (MO); WP-Li: WP + Lecithin and Propionic Acid (LPA); WP-Si: WP + Alkylene Oxide (AO); WP-Di: WP Formulation; WP-Ta: WP + Sodium Lauryl Ether Sulfate (SLES); WP-Au: WP + Soybean Oil Methyl Ester (SOME); WP-Ag: WP + Nonylphenoxy Polyethanol (NPPE); WP-In: WP + Nonylphenol Ethoxylate (NPE); SC-Ni: SC + Mineral Oil (MO); SC-Li: SC + Lecithin and Propionic Acid (LPA); SC-Si: SC + Alkylene Oxide (AO); SC-Di: SC Formulation; SC-Ta: SC + Sodium Lauryl Ether Sulfate (SLES); SC-Au: SC + Soybean Oil Methyl Ester (SOME); SC-Ag: SC + Nonylphenoxy Polyethanol (NPPE); SC-In: SC + Nonylphenol Ethoxylate (NPE). *Capital letters are used when comparing differences between formulations. Lowercase letters are used when comparing differences between treatments within each formulation. Comparisons followed by the same letter did not differ in Tukey’s t test at P = 0.05. Source: Author.

When mixtures of the concentrated suspension (SC) formulation were compared, the wettable powder (WP) formulation had the highest VMD values. When adjuvants were added, VMD remained the same in the Bt WP mixture. Only the addition of mineral oil (MO) resulted in a higher VMD in the Bt SC (Fig. 1). The highest SPAN values were observed in the treatments with lecithin- and propionic-acid-based adjuvants (LPA), organosilicon (AO) and nonylphenol ethoxylate (NPE) in the WP formulation, and MO in the Bt SC mixture. Among the formulations, mixtures composed of nonylphenoxy polyethanol (NPPE) and NPE in the Bt WP had the highest SPAN values, while the Bt SC mixtures, obtained the highest SPAN values when MO, NPPE, and NPE adjuvants were added (Fig. 1). The SC formulation mixtures had higher drift risk (%V < 100 µm). In both formulations, the addition of vegetable oil (SOME), NPPE, and NPE adjuvants led to higher %V < 100 µm values (Fig. 1).

### Surface tension and contact angle

**Figure 2:**
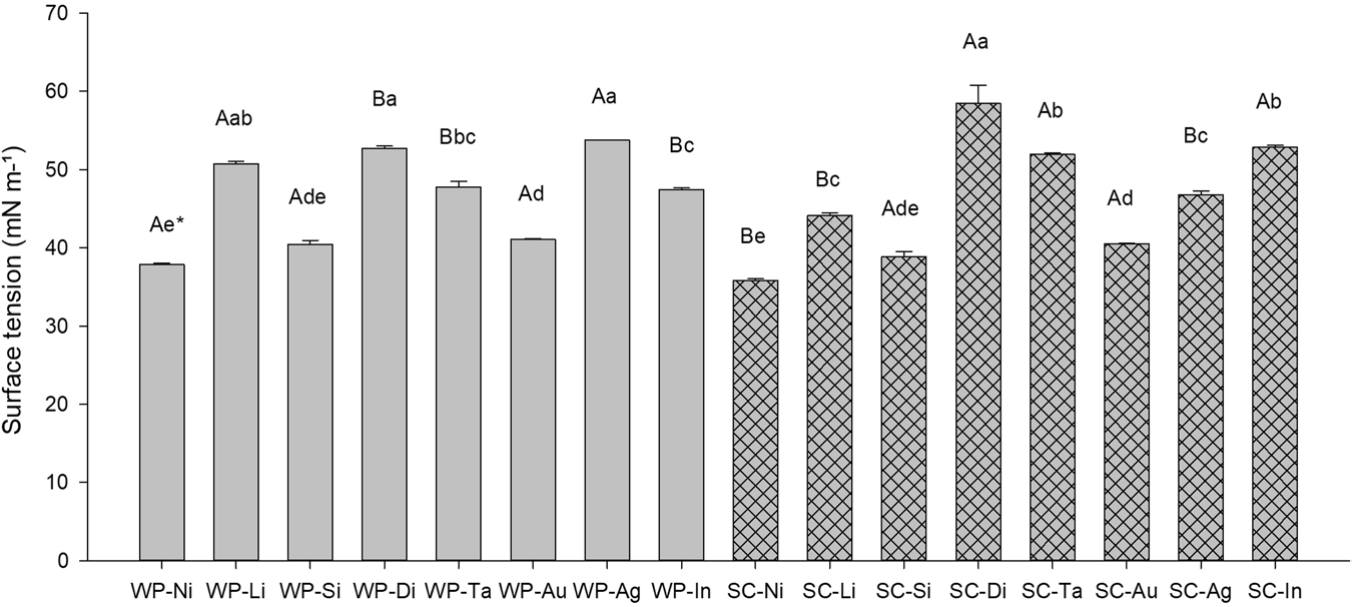
Surface tension values in the different mixtures at 5 seconds. WP-Ni: WP + Mineral Oil (MO); WP-Li: WP + Lecithin and Propionic Acid (LPA); WP-Si: WP + Alkylene Oxide (AO); WP-Di: WP Formulation; WP-Ta: WP + Sodium Lauryl Ether Sulfate (SLES); WP-Au: WP + Soybean Oil Methyl Ester (SOME); WP-Ag: WP + Nonylphenoxy Polyethanol (NPPE); WP-In: WP + Nonylphenol Ethoxylate (NPE); SC-Ni: SC + Mineral Oil (MO); SC-Li: SC + Lecithin and Propionic Acid (LPA); SC-Si: SC + Alkylene Oxide (AO); SC-Di: SC Formulation; SC-Ta: SC + Sodium Lauryl Ether Sulfate (SLES); SC-Au: SC + Soybean Oil Methyl Ester (SOME); SC-Ag: SC + Nonylphenoxy Polyethanol (NPPE); SC-In: SC + Nonylphenol Ethoxylate (NPE). *Capital letters are used when comparing differences between formulations. Lowercase letters are used when comparing differences between treatments within each formulation. Comparisons followed by the same letter do not differ in Tukey’s test at P = 0.05. Source: Author.

**Figure 3:**
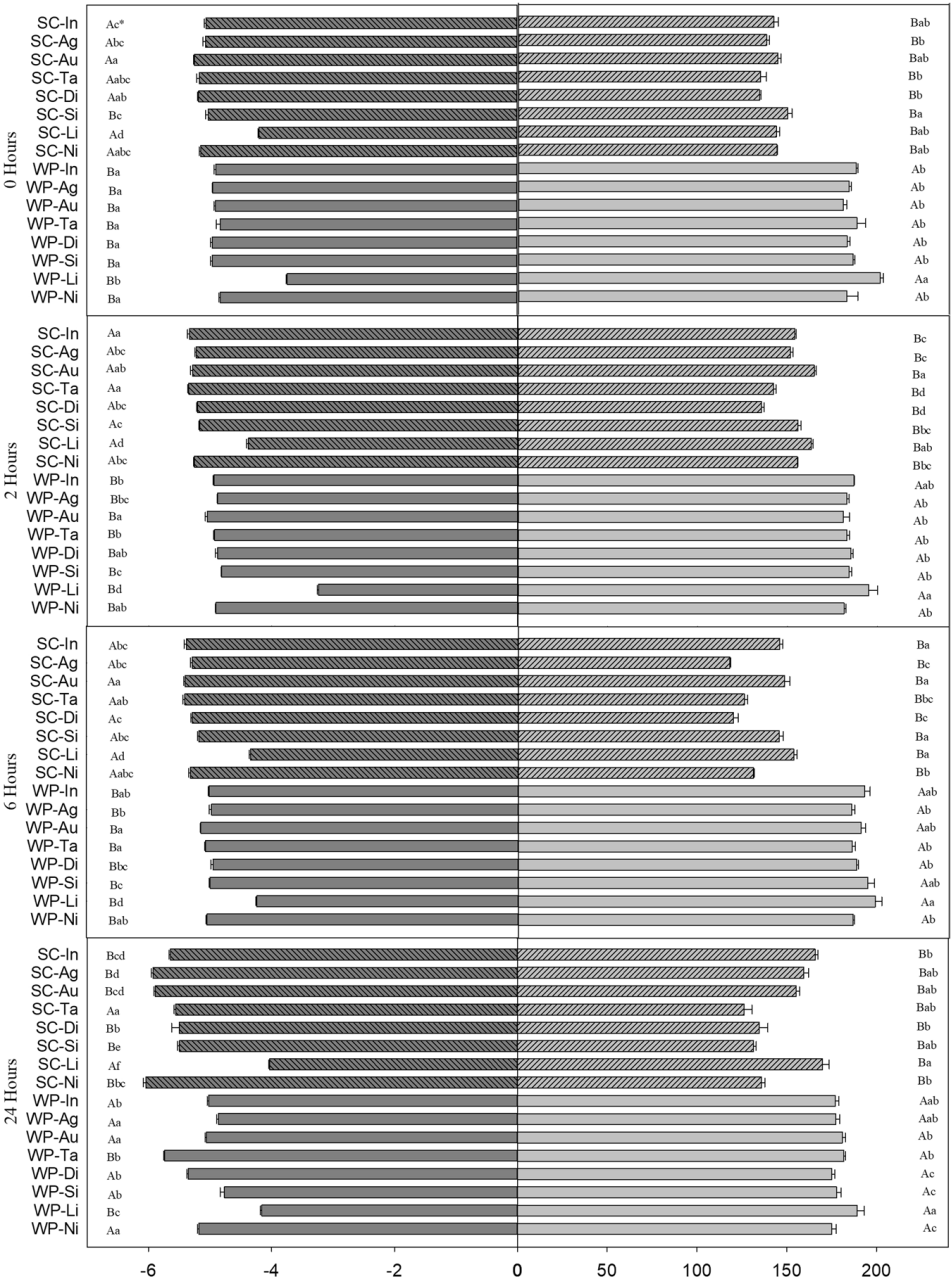
Potential of Hydrogen (Left) and Electric Conductivity (Right) in the different rest periods after agitation. Legend: SC-Ni: SC + Mineral Oil (MO); SC-Li: SC + Lecithin and Propionic Acid (LPA); SC-Si: SC + Alkylene Oxide (AO); SC-Di: SC Formulation; SC-Ta: SC + Sodium Lauryl Ether Sulfate (SLES); SC-Au: SC + Soybean Oil Methyl Ester (SOME); SC-Ag: SC + Nonylphenoxy Polyethanol (NPPE); SC-In: SC + Nonylphenol Ethoxylate (NPE); WP-Ni: WP + Mineral Oil (MO); WP-Li: WP + Lecithin and Propionic Acid (LPA); WP-Si: WP + Alkylene Oxide (AO); WP-Di: WP Formulation; WP-Ta: WP + Sodium Lauryl Ether Sulfate (SLES); WP-Au: WP + Soybean Oil Methyl Ester (SOME); WP-Ag: WP + Nonylphenoxy Polyethanol (NPPE); WP-In: WP + Nonylphenol Ethoxylate (NPE). *Capital letters are used when comparing differences between formulations. Lowercase letters are used when comparing differences between treatments within each formulation. Comparisons followed by the same letter do not differ in Tukey’s test at P = 0.05. Source: Author

Addition of MO, AO, and SOME adjuvants to the WP and SC mixtures resulted in the lowest surface stresses, while the highest stresses were observed in Bt WP mixtures and when the SLES adjuvant was added (Fig. 2). The lowest surface stresses among the formulations were observed in OM, LPA, Bt SC, and NPPE mixtures, respectively, while the highest were observed in SLES and NPE mixtures (Fig. 3). In spray solutions composed of Bt WP, the lowest surface stresses were obtained where MO, AO and SOME adjuvants had been added, while the highest were observed in Bt without adjuvants and with added NPPE and LPA adjuvants (Fig. 2).

Droplet contact angle differed for the two evaluated surfaces; the parafilm surface had higher values than the cotton leaf surface (Table 2). On the cotton leaf, the WP formulation had the lowest contact angles when compared to BS. On the parafilm surface, WP treatments with added LPA, AO, and NPPE adjuvants had higher contact angles, as did the SC without adjuvant and the SLES and NPE adjuvants (Table 2). Comparing the spray solutions in each formulation, the LPA in the WP formulation on the leaf surface had the greatest angle, whereas the Bt WP spray solution without adjuvant was greater on the parafilm surface. In the SC formulation, the addition of LPA and NPE provided greater contact angles of droplets on the sheet surface. In the parafilm, the Bt SC without adjuvant and with NPE had the highest values of droplet contact angle values (Table 2).

**Table 1:** Commercial products used to evaluate physical and chemical characteristics of mixtures in WP and SC formulations of Dipel in terms of droplet size spectrum, contact angle, surface tension of droplets and hydrogen and electrical conductivity potential of the mixtures.

Mixtures*	Dosages	a.i of products
Dipel^®^ WP	700 g ha^−1^	*Bacillus thuringiensis*, var. kurstaki
Dipel^®^ WP + In	700 g ha^−1^ + 0.2% v/v	Bt + Nonylphenol Ethoxylate (NPE)
Dipel^®^ WP + Ag	700 g ha^−1^ + 0.2% v/v	Bt + Nonylphenoxy Polyethanol (NPPE)
Dipel^®^ WP + Li	700 g ha^−1^ + 0.2% v/v	Bt + Lecithin and Propionic Acid (LPA)
Dipel^®^ WP + TA	700 g ha^−1^ + 0.2% v/v	Bt + Sodium Lauryl Ether Sulfate (SLES)
Dipel^®^ WP + Ni	700 g ha^−1^ + 750 mL ha^-1^	Bt + Mineral Oil (MO)
Dipel^®^ WP + Si	700 g ha^−1^ + 0.2% v/v	Bt + Alkylene Oxide (AO)
Dipel^®^ WP + Au	700 g ha^−1^ + 375 mL ha^−1^	Bt + Soybean Oil Methyl Ester (SOME)
Dipel^®^ SC	625 mL ha^−1^	*Bacillus thuringiensis*, var. kurstaki
Dipel^®^ SC + In	625 mL ha^−1^ + 0.02% v/v	Bt + Nonylphenol Ethoxylate (NPE)
Dipel^®^ SC + Ag	625 mL ha^−1^ + 0.02% v/v	Bt + Nonylphenoxy Polyethanol (NPPE)
Dipel^®^ SC + Li	625 mL ha^−1^ + 0.02% v/v	Bt + Lecithin and Propionic Acid (LPA)
Dipel^®^ SC + TA	625 mL ha^−1^ + 0.02% v/v	Bt + Sodium Lauryl Ether Sulfate (SLES)
Dipel^®^ SC + Ni	625 mL ha^−1^ + 750 mL ha^−1^	Bt + Mineral Oil (MO)
Dipel^®^ SC + Si	625 mL ha^−1^ + 0.02% v/v	Bt + Alkylene Oxide (AO)
Dipel^®^ SC + Au	625 mL ha^−1^ + 375 mL ha^−1^	Bt + Soybean Oil Methyl Ester (SOME)

*All the dosages are recommended by the manufacturers for a mixture volume of 150 L ha^−1^. a.i: Active ingredient. Source: Author.

**Table 2:** Values of droplet contact angle for the mixture in WP and SC formulations on cotton leaf and parafilm surfaces.

Treatments		Surface	
		Leaf	Parafilm
Formulation	WP + Ni	53.13 Bbe*	73.70 Aad
	WP + Li	74.91 Bba	88.20 Aab
	WP + Si	64.73 Babc	88.10 Aab
	WP	57.32 Bbcde	99.17 Aba
	WP + Ta	53.62 Bbde	86.57 Abc
	WP + Au	59.06 Bbcde	79.44 Aacd
	WP + Ag	68.78 Bbab	88.76 Aab
	WP + In	61.49 Bbbcd	88.82 Abb
	SC + Ni	59.10 Bae	74.73 Aae
	SC + Li	82.52 Baab	92.20 Aab
	SC + Si	61.68 Bade	79.35 Abde
	SC	69.43 Bacd	107.12 Aaa
	SC + Ta	71.30 Bac	96.26 Aab
	SC + Au	72.50 Bac	83.55 Aacd
	SC + Ag	75.86 Babc	90.72 Aabc
	SC + In	84.24 Baa	105.77 Aaa

Legend: WP-Ni: WP + Mineral Oil (MO); WP-Li: WP + Lecithin and Propionic Acid (LPA); WP-Si: WP + Alkylene Oxide (AO); WP-Di: WP Formulation; WP-Ta: WP + Sodium Lauryl Ether Sulfate (SLES); WP-Au: WP + Soybean Oil Methyl Ester (SOME); WP-Ag: WP + Nonylphenoxy Polyethanol (NPPE); WP-In: WP + Nonylphenol Ethoxylate (NPE); SC-Ni: SC + Mineral Oil (MO); SC-Li: SC + Lecithin and Propionic Acid (LPA); SC-Si: SC + Alkylene Oxide (AO); SC-Di: SC Formulation; SC-Ta: SC + Sodium Lauryl Ether Sulfate (SLES); SC-Au: SC + Soybean Oil Methyl Ester (SOME); SC-Ag: SC + Nonylphenoxy Polyethanol (NPPE); SC-In: SC + Nonylphenol Ethoxylate (NPE). *Capital letters in the row are used when comparing differences between surfaces in the different treatments and formulations. Lowercase letters in the column compare differences between formulations within treatments. Underlined lowercase letters are used when comparing differences between treatments within each formulation. Comparisons followed by the same letter do not differ in Tukey’s test at P = 0.05. Source: Author.

### Potential of hydrogen (pH) and electrical conductivity (E.C.)

The mixtures were subjected to four different periods of rest after agitation for evaluation of hydrogenionic potential (pH) and electrical conductivity (E.C.). During all rest periods, the highest E.C. values were observed when the LPA adjuvant was added to the SC and WP formulations of Bt bioinsecticide. The mixtures with the SC formulation had the highest pH values and the lowest E.C. values, while the WP spray solutions had the lowest pH values and the highest E.C. The pH and E.C. did not significantly vary during the rest periods of the mixture (Fig. 3).

## Discussion

The Dipel® WP formulation mixtures had higher volume median diameter (VMD) and consequently, lower susceptibility to drift (%V < 100 µm) and evaporation potential, reducing dehydration-induced non-viability of bacterial spores and providing greater protection against direct exposure to ultraviolet rays^21^. Larger droplets may support better persistence and survival of Bt in unfavorable environmental conditions like hot weather, because they are composed of more water and spores. Very fine droplets such as those with VMD less than 100 µm are highly susceptible to drift, and evaporate more easily when air humidity is below 55%^22^.

For this reason, the mixture with Dipel® SC formulation had less uniform droplets (SPAN) than the mixture with Dipel® WP. SPAN indicates uniformity of droplet diameter; the closer to zero, the greater the uniformity and the smaller the variation in diameter in relation to VMD. These mixtures therefore have greater droplet uniformity, allowing for applications with droplet diameter closer to the target. Mixtures with variations between small and large droplets are more prone to loss by drift and runoff, respectively^23^. Regarding drift susceptibility, Bt WP mixtures were less likely to have losses. The lower percentage of volumes with droplets less than 100 µm, the lower the potential drift during the application of Bt, because droplets that are more prone to drift are more affected by meteorological phenomena^24^. Size differences observed among droplets of the bioinsecticide formulations used in this study can be attributed to the formulation composition, because commercial product components affect the spectrum of droplets formed during spraying^25^. In this study, the SC formulation was more susceptible to drift than the WP formulation.

Adding adjuvants reduced the surface tension of the Bt SC and WP droplets. In both formulations, the mixtures containing MO, SOME and AO had the lowest surface tension values. Decreased mixture surface tension may be related to leaf spreading. However, correlation depends on the affinity of the liquid to the plant species on which the droplet is deposited. Notably, differences in the epidermis, such as composition of cuticular wax and cell anatomy among plant species, may affect spreading^26,27^.

Similar results were found for the Bt mixture when mineral and vegetable oil were added, reducing the surface tension of the spray droplets and resulting in greater spreading of the mixture in cassava leaves (*Manihot esculenta* Crantz)^7^. Surface tension was reduced with the addition of oils because of micelle formation by the molecules in the mixture^28,29^. These bonds act as a bridge between water molecules and insecticide, reducing the surface tension of the mixture and replacing stronger bonds represented by hydrogen bridges in the water^16^. Higher surface tension values were observed in NPPE, NPE, SLES, Bt WP and Bt SC mixtures, indicating decreased spreading power on the leaf surface. Droplets with higher surface tension, therefore, have higher VMD and are less susceptible to drift because solutions with higher surface tension are able to produce thicker droplets. These droplets are also more resistant to wind during spraying^30^.

The smallest droplet contact angles were observed in the WP formulation mixtures for both the parafilm and the cotton leaf. The mixtures with this formulation had higher spreading capacity on the plant leaf surfaces. Thus, the lower the surface tension of the liquid, the lower the contact angle. This is because the addition of surfactants promote molecular rearrangements such that the polar end of the molecules turns toward the water, while the other end turns to the interface where liquid contacts the surface, thus “breaking” the surface tension of the mixture, reducing the contact angle in relation to the leaf and increasing wettability^28^.

Adding of LPA to Bt mixtures decreased pH and increased electrical conductivity during all rest periods. During vegetative growth, acidic media provides greater sporulation in a Bt^12^-based mixture because the bacterium produces and excretes in the pyruvate and acetate culture medium resulting from carbohydrate fermentation^31^. At the end of vegetative growth, the synthesis of poly-β-hydroxybutyrate (PHB) begins, serving as an intracellular reserve of carbon and energy for sporulation in many *Bacillus* spp.^13^. The synthesis of PHB begins once the minimum pH value is reached, and lasts for a few hours until reaching the maximum concentration, when sporulation begins^32^. LPA contains lecithin and propionic acid, which influence the production of Bt spores, because synthesizing protein crystals requires a high amount of energy^13^. These components are an important energy source for crystal synthesis, contributing to the growth of colonies both regarding quantity and force.

The electrical conductivity of the mixtures remained constant during the rest period. The WP formulation had the highest E.C. values. The difference in electrical conductivity can be attributed to the composition of the mixture, which alters the biological efficacy of crop protection products^33^. It is important to study pH and E.C. because these factors can accelerate degradation of crop protection products influencing the rest of the application^34^.

## Conclusions

The Bt WP formulation, with and without addition adjuvants, performed better with regard to droplet size and droplet spreading on the plant surface because its larger droplets made it less susceptible to drift. The lecithin and propionic acid-based adjuvant addition had slightly acidic pH and high electrical conductivity in the mixtures, which are physical and chemical characteristics favorable to the production of crystals and Bt spores during the fermentation process because they act as an energy source.

## Methods

### Spray droplet size spectrum

The droplet size was determined by laser diffraction using a particle diameter meter (Mastersizer S® version 2.19). An optical unit in the equipment determines the droplet diameter of the sprayed spectrum by the path deviation of the laser when reaching them. The smaller the particle, the greater the degree of diffraction experienced by the ray of light^35^. The treatments are described in Table 1 as the Concentrated Suspension (SC) and Wettable Powder (WP) formulations, and a XR11003 (TeeJet Technologies) hydraulic deflector jet spray tip model was used for testing all spray solutions. The spray was triggered by compressed air and the pressure was constant at 275 kPa with a precision pressure regulator for each tip. Three specimens of the tip were evaluated four times, totaling 12 repetitions per treatment. The design was random and the values of VMD, SPAN and percentage of volume with droplets smaller than 100 μm (%V < 100 µm) were evaluated.

### Surface tension and droplet angle on cotton leaf surface

The mixture surface tension was measured on a section of cotton leaf and parafilm, 0.5 cm wide × 5 cm long. Surface tension was determined using the hanging droplet method with an automatic tensiometer (Model OCA 15 Plus, Dataphysics Germany). The image of the droplet formed at the end of a syringe is captured by a camera and the equipment analyzes the shape of the droplet by axis asymmetry (ADSA-Axisymmetric Droplet Shape Analysis). Digitizing and analyzing the droplet profile determined surface tension, using the Young-Laplace equation for adjustment^36^.

### Mixture stability, pH, and electrical conductivity

Lidded 250 mL beakers were used for the preparation of the mixtures using the recommended concentration of each product (Table 1). The liquid products were dosed using a 1,000 graduated pipette μL (LabMate®). The pH (pHmeter QUIMIS® Q400AS) and electrical conductivity (Condutivimeter Mars® MB-11P) were measured before and after mixing. The mixture was prepared and evaluated in a temperature of 25 ± 2 °C and relative humidity of 65 ± 5%.

The mixtures were evaluated at the following intervals: immediate separation after mixing (0) and after 2, 6, and 24 hours of the rest period, The effects of interactions between the products were analyzed for homo- and heterogeneity (flocculation, sedimentation, phase separation, formation of lumps and formation of crystals) and foam formation^37^. A metal sieve with a nominal opening of 149 μm was used^38^ to evaluate flocculation and formation of lumps and crystals. Sedimentation was measured by the volume of mixture deposited at the bottom of the beaker.

### Statistical analysis

The droplet size spectrum and surface tension were analyzed using a 2 × 8 factorial design (Dipel® formulation versus adjuvants). The contact angle was analyzed using a 2 × 8 × 2 factorial design (Dipel® formulation versus adjuvants versus surface), and the pH and E.C. values were analyzed using a 2 × 8 × 4 factorial design (Dipel® formulation versus adjuvants versus reading time). The data obtained were subjected to analysis of variance, and when significant, the means were compared using the Tukey test at P = 0.05 using SAS USER 9.3 software^39^.

## Data Availability

The datasets generated during and/or analysed during the current study are available from the corresponding author on reasonable request.
